# Neuroinflammation and Its Association with Cognition, Neuronal Markers and Peripheral Inflammation after Chemotherapy for Breast Cancer

**DOI:** 10.3390/cancers13164198

**Published:** 2021-08-20

**Authors:** Gwen Schroyen, Jeroen Blommaert, Donatienne van Weehaeghe, Charlotte Sleurs, Mathieu Vandenbulcke, Nina Dedoncker, Sigrid Hatse, An Goris, Michel Koole, Ann Smeets, Koen van Laere, Stefan Sunaert, Sabine Deprez

**Affiliations:** 1 Leuven Brain Institute, KU Leuven, 3000 Leuven, Belgium; jeroen.blommaert@kuleuven.be (J.B.); charlotte.sleurs@kuleuven.be (C.S.); mathieu.vandenbulcke@kuleuven.be (M.V.); nina.dedoncker@kuleuven.be (N.D.); an.goris@kuleuven.be (A.G.); koen.vanlaere@kuleuven.be (K.v.L.); stefan.sunaert@kuleuven.be (S.S.); sabine.deprez@kuleuven.be (S.D.); 2 Leuven Cancer Institute, KU Leuven, 3000 Leuven, Belgium; sigrid.hatse@kuleuven.be (S.H.); michel.koole@kuleuven.be (M.K.); ann.smeets@kuleuven.be (A.S.); 3 Department of Imaging and Pathology, KU Leuven, 3000 Leuven, Belgium; 4 Department of Oncology, KU Leuven, 3000 Leuven, Belgium; 5 Department of Nuclear Medicine and Molecular Imaging, KU Leuven, 3000 Leuven, Belgium; donatienne.vanweehaeghe@kuleuven.be; 6 Nuclear Medicine and Molecular Imaging, University Hospitals Leuven, 3000 Leuven, Belgium; 7 Department of Neurosciences, KU Leuven, 3000 Leuven, Belgium; 8 Psychiatry, University Hospitals Leuven, 3000 Leuven, Belgium; 9 Surgical Oncology, University Hospitals Leuven, 3000 Leuven, Belgium; 10 Radiology, University Hospitals Leuven, 3000 Leuven, Belgium

**Keywords:** neuroimaging, breast cancer, chemotherapy, neuroinflammation, PET-MR

## Abstract

**Simple Summary:**

Up to 70% of chemotherapy-treated patients experience problems with memory and concentration, potentially caused by direct and indirect neurotoxicity, such as (neuro-)inflammatory processes. Can neuroinflammation changes be detected in chemotherapy-treated patients with breast cancer using translocator protein [^18^F]DPA714 simultaneous positron emission tomographic- and magnetic resonance imaging? Moreover, what is the association with clinical biomarkers? In a study including 19 chemotherapy-treated breast cancer patients, 18 chemotherapy-naïve and 37 healthy controls, we found significant relative glial overexpression in parietal and occipital brain regions in chemotherapy-treated patients compared to controls, which were associated with cognitive abnormalities and markers of neuronal survival. Shortly after ending chemotherapy, changes in brain neuroinflammation seem to occur, possibly contributing to the cognitive decline seen in breast cancer patients. Additionally, blood levels of an axonal damage marker were 20-fold higher in chemotherapy-treated patients, providing evidence for its use as a biomarker to assess neurotoxic effects of anticancer chemotherapies.

**Abstract:**

To uncover mechanisms underlying chemotherapy-induced cognitive impairment in breast cancer, we studied new biomarkers of neuroinflammation and neuronal survival. This cohort study included 74 women (47 ± 10 years) from 22 October 2017 until 20 August 2020. Nineteen chemotherapy-treated and 18 chemotherapy-naïve patients with breast cancer were assessed one month after the completion of surgery and/or chemotherapy, and 37 healthy controls were included. Assessments included neuropsychological testing, questionnaires, blood sampling for 17 inflammatory and two neuronal survival markers (neurofilament light-chain (NfL), and brain-derived neurotrophic factor (BDNF) and PET-MR neuroimaging. To investigate neuroinflammation, translocator protein (TSPO) [^18^F]DPA714-PET-MR was acquired for 15 participants per group, and evaluated by volume of distribution normalized to the cerebellum. Chemotherapy-treated patients showed higher TSPO expression, indicative for neuroinflammation, in the occipital and parietal lobe when compared to healthy controls or chemotherapy-naïve patients. After partial-volume correction, differences with healthy controls persisted (*p*_FWE_ < 0.05). Additionally, compared to healthy- or chemotherapy-naïve controls, cognitive impairment (17–22%) and altered levels in blood markers (*F* ≥ 3.7, *p* ≤ 0.031) were found in chemotherapy-treated patients. NfL, an axonal damage marker, was particularly sensitive in differentiating groups (*F* = 105, *p* = 4.2 × 10 ^−21^), with levels 20-fold higher in chemotherapy-treated patients. Lastly, in chemotherapy-treated patients alone, higher local TSPO expression was associated with worse cognitive performance, higher blood levels of BDNF/NfL, and decreased fiber cross-section in the corpus callosum (*p*_FWE_ < 0.05). These findings suggest that increased neuroinflammation is associated with chemotherapy-related cognitive impairment in breast cancer. Additionally, NfL could be a useful biomarker to assess neurotoxic effects of anticancer chemotherapies.

## 1. Introduction

With advances in cancer treatment, the number of cancer survivors has grown remarkably [1]. Emphasis has gone to understanding how cancer treatment can impact survivors’ quality of life. Cancer-related cognitive impairment (CRCI) is broadly reported, with up to 70% of patients being affected, mainly in memory and attention, especially related to chemotherapy [2]. CRCI can emerge before the start of therapy, during, or persist up to years after treatment [3]. While underlying mechanisms remain largely unknown, CRCI is described as a complex interaction of vulnerability [4], cancer biology, aging [5,6], and both direct (i.e., cytostatics crossing the blood-brain barrier (BBB) [7]) or indirect toxic treatment effects (i.e., cytokine-induced neuroinflammation, hormonal deregulation, or oxidative damage [8,9]). 

After cancer treatment, increased pro-inflammatory cytokines are found in patients [10], which can cross and disrupt the BBB, activate microglia or astrocytes, and potentially increase permeability for cytotoxic agents [9]. Interestingly, peripheral cytokine levels are found to correlate with cognitive changes [3,11]. Moreover, rodent studies have demonstrated changed microglial levels after chemotherapy [12,13,14,15]. These findings suggest induced neuroinflammation as an interesting pathway to explain acute behavioral effects seen after chemotherapy, sometimes transitioning to chronic syndromes [16].

In vivo assessment of inflammatory processes is made possible by positron emission tomography (PET) using radiolabeled ligands selective for the 18kDa translocator protein (TSPO) [17,18]. Minimally expressed in the healthy brain, TSPO is overexpressed when microglia and astrocytes are activated, and is presumed to serve as a marker for neuroinflammation [17,19]. TSPO-imaging could therefore be extremely valuable to evaluate neuroinflammation as a causative factor for chemotherapy-induced cognitive impairment.

Besides indirect toxicity, chemotherapeutics could more directly induce neuronal brain damage. In rodents, cytostatics increase apoptosis and reduce neurogenesis [20,21]. Promising markers to investigate neurotoxicity in humans are neurofilaments, which demonstrate high prognostic and diagnostic accuracy for neurodegenerative diseases, such as amyotrophic lateral sclerosis [22] and Alzheimer’s disease [23], even in their earlier stages. Moreover, neurofilaments increase in a chemotherapy dose-dependent manner in breast cancer patients, suggesting their potential as a marker for neuronal damage after chemotherapy [24].

In vivo measurement of neural microstructure and function can be assessed by magnetic resonance imaging (MRI). Since oligodendrocytes, the myelin forming cells of the central nervous system (CNS), are known to be especially vulnerable to cytostatics [25,26], diffusion-weighted MRI is used to study white matter (WM)-microstructure in vivo. Chemotherapy-induced changes in WM-microstructure are associated with cognitive decline in breast cancer patients [27,28,29]. Additionally, rodent research has shown that neuroinflammation after chemotherapy can modulate myelin structure and myelination [30]. However, such interactions remain to be elucidated in a clinical setting.

This study aimed at investigating (in) direct neurotoxicity after chemotherapy for breast cancer. Specifically, we investigated neuroinflammation and its association with cognitive decline, blood markers of neuronal survival and inflammation, and WM-microstructure.

## 2. Patients and Methods

This prospective cohort study enrolled participants from October 2017 until August 2020. Women < 65 years diagnosed with early-stage breast cancer were contacted at the University Hospitals Leuven. Patients treated with chemotherapy (C+, four rounds of epirubicin 90 mg/m^2^ + cyclophosphamide 600 mg/m^2^ and four to 12 rounds of paclitaxel 80 mg/m^2^) and control patients not scheduled for chemotherapy (C−) were included. Healthy controls (HC) were recruited through online advertisements. Both control groups, C− and HC, were matched at group level to the C+ group based on age and education. Women with MRI/PET-contraindications, (history of) cancer treatment, psychiatric/neurological condition/injury, mental retardation, or systemic steroid use, were excluded. Via blood collection, participants were genetically screened for the TSPO single-nucleotide polymorphism rs6971, determining [^18^F]DPA714-tracer affinity. Low-affinity (LA) binders were excluded [31] (supplementary materials (SM)). Data collection took place post-chemotherapy or post-surgery, before the start of radiotherapy and/or antihormone therapy. Detailed information of the included cohort is provided in Table 1.

### 2.1. Neuroimaging

Details can be found in SM.

Participants underwent simultaneous [^18^F]DPA714-PET-MR neuroimaging on a GE-SIGNA scanner (GE Healthcare, Milwaukee). After a bolus injection of [^18^F]DPA714 (144 ± 16 MBq, 388 ± 252 GBq/µmol), 60-min dynamic PET scans were acquired in list-mode, during which 5 mL arterial samples were manually collected to derive the arterial input-curve and parent-free fraction. Simultaneously, a 3-dimensional T1-weighted, zero-echo-time for attenuation correction [32] and multi-shell-diffusion MR images were acquired.

For the quantification of [^18^F]DPA714-PET, voxel-based and volume-of-interest (VOI) analyses were performed using Logan graphical analysis (LGA) [33] with total distribution volume (V_T_) as the main parameter of interest. VOIs included frontal, temporal, occipital, parietal, insular, and cingulate cortices, amygdala, hippocampus, thalamus, striatum, cerebellum, and WM. To account for brain atrophy, voxel-based morphometry (VBM) analysis on T1-weighted MRI and region-based voxel-wise partial-volume correction (PVC) [34] was performed on V_T_-images (Freesurfer v6). To correct for genotype, voxel and VOI V_T_ were i) analyzed with a binding-affinity covariate (high-affinity/medium-affinity, HA/MA) and ii) V_T-ratios_ were calculated by dividing each voxel/VOI by the corresponding mean cerebellar V_T_. The cerebellum serves as a pseudo-reference region, as seen in previous studies in “middle-aged” individuals [17]. Earlier structural/metabolic neuroimaging studies mainly observed chemotherapy-induced changes in frontal/temporal regions, suggesting the cerebellum to be less affected and a possible reference region [35,36].

Fixel-based analysis of diffusion-weighted images (MRtrix v3.0) [37,38,39] was used to study WM micro- and macrostructure. This novel technique addresses the complexity of crossing fibers, in contrast to older techniques such as diffusion-tensor imaging [37], providing measures of fiber density (FD), fiber cross-section (FC), and a combined measure of fiber density and cross-section (FDC).

### 2.2. Clinical Parameters

Cognitive functioning was assessed using nine neuropsychological tests covering memory, attention/concentration, processing speed and executive functioning, following ICCTF guidelines [40], from which a Global Deficit Score (GDS) [41] was derived (GDS ≥ 0.50 indicates cognitive impairment) [41,42]. Participants completed questionnaires evaluating anxiety [43], depression [44], stress [45], fatigue [46], and cognitive failure (CFQ-total > 55 indicates severe cognitive complaints) (SM) [47,48]. 

Neurofilament light chain (NfL) was assessed with an enzyme-linked immunosorbent-kit (UmanDiagnostics, Umea) on serum [49] and quantified with an electrochemiluminescent assay [50]. Mean values across triplicates were used for analysis. We explored the diagnostic cut-off of 26.6 pg/mL for NfL, which has been proposed for neurodegenerative disease with a 91% sensitivity using the same immunoassay [50]. Inflammatory markers and brain-derived neurotrophic factor (BDNF) were determined by bead-based-immunoassay (ImTec Diagnostics, Antwerp and YSL AimPlex, BioLegend, San Diego) on plasma (SM).

### 2.3. Statistics

Clinical and PET-VOI data were assessed for normality (log-transformed when necessary) and compared between groups, using SPSS 27.0 (Chicago, IL, USA). Chi-square tests and one-way analyses of variance were performed to evaluate group differences in categorical and numerical variables, respectively, with post-hoc least-significance difference to assess which groups differ. Statistical significance was inferred at *p* < 0.05, with age as covariate for blood markers and years of education for cognition.

T1w-modulated GM and PVC-V_T_ maps were analyzed with SPM12 and diffusion-derived fixels with MRtrix 3.0, using a generalized linear model to explore group differences. Absolute-V_T_ analysis was corrected for binding affinity, MRI analyses for age and intracranial volume (ICV) when relevant (T1w, FDC and FC). For C+ patients, linear regression models (SPM12) explored associations between whole-brain PET-PVC-V_T-ratio_ and cognition (GDS and CFQ; corrected for yes/no higher education), or blood markers (NfL, BDNF and one comprehensive measure for peripheral inflammation; corrected for age). To reduce the number of variables, (i) only PET-PVC images showing group differences were used and (ii) principal component (PC) analysis was performed for the whole sample on those inflammatory markers showing group differences, to extract the first PC (inflammatory markers are expected to interact in a network [51]). The relationship of PVC-V_T-ratio_ with whole-brain WM-morphometry (FD, corrected for age; log FC and FDC, corrected for age and ICV) was also explored using regression models (MRtrix). To reduce the number of variables, only the V_T-ratio_ of the region showing an association with cognition (frontal) was used for these analyses. 

Statistical significance for image analyses was inferred with a height threshold of *p_uncorrected_* < 0.001 and a cluster-level familywise-error correction for multiple comparisons (FWE) of *p_FWE_* < 0.05, while a threshold of height *p_uncorrected_* < 0.005 and cluster *p*_FWE_ < 0.05 was used for the group PET analyses. No correction for multiple comparisons was performed for other analyses seen the explorative character of this study.

## 3. Results

### 3.1. Participants

**Figure 1:**
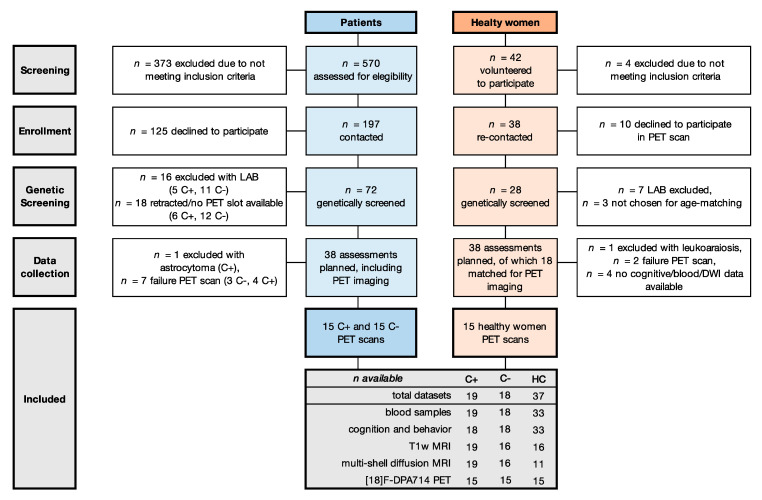
**Flow diagram of included participants.** Abbreviations: C− = chemotherapy-naïve breast cancer patients, C+ = breast cancer patients treated with chemotherapy, DWI = diffusion weighted imaging, HC = healthy controls, LAB = low affinity binder.

Of 197 eligible patients, 54 provided informed consent (27%). Of those, 16 were excluded because of LA binding [31] and one because of an incidental CNS tumor (Figure 1). The final sample included 19 chemotherapy-treated patients (C+), 18 chemotherapy-naïve patients (C−) and 37 healthy controls (HC). Demographics and medical data are provided in Table 1. Groups showed no differences regarding age, education, or BMI. A difference was found for menopausal status, related to chemotherapy-induced menopause for C+ [52], and days since last treatment, since assessments were required before the start of radiotherapy, which occurred sooner for C+ than C−.

### 3.2. Neuroimaging

VBM revealed no GM atrophy for patients compared to HC. Global VOI and voxel-level analysis showed no group differences in LGA absolute V_T_, both for uncorrected and PVC values. Cerebellar V_T_ did not differ between groups (*p* = 0.51, *F* = 0.69) and showed the lowest V_T_ of cortical GM VOIs (Supplementary Table S2), supporting its use as pseudo-reference region.

**Figure 2:**
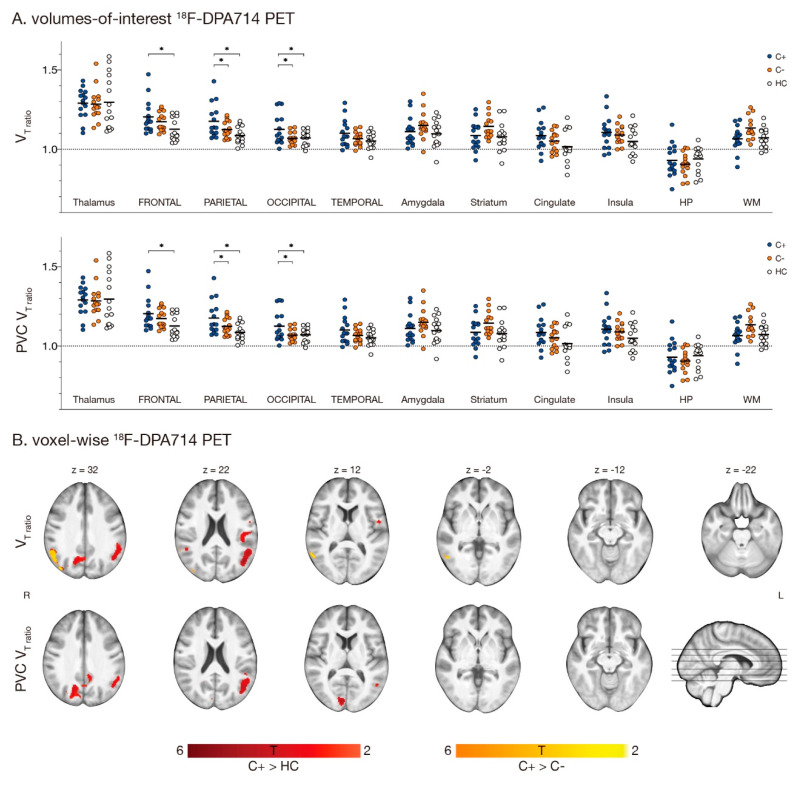
**Regions showing [^18^F]DPA714 V_T-ratio_ differences with LGA.** Fifteen chemotherapy-treated patients (C+) were assessed for [^18^F]DPA714 V_T-ratio_ and compared to 15 chemotherapy-naïve patients (C−) and 15 healthy women (HC). (**A**) Volumes-of-interest based logan-graphical analysis (LGA) results of 11 volumes of interest are presented, showing higher V_T-ratio_ in C+ patients compared to HC in the parietal lobe. After partial volume correction (PVC), C+ patients showed higher V_T-ratio_ compared to C− and HC in the parietal and occipital lobe and additionally in the frontal lobe when compared to HC (* *p* < 0.05). (**B**) Voxel-based whole brain LGA results are presented, showing higher V_T_-_ratio_ in C+ patients compared to HC (blue) and C− patients (orange) in the occipital and parietal lobe. After PVC, only differences between C+ and HC persisted for V_T-ratio_ images (all *p_uncorrected_* < 0.005, *p_cluster FWE-corrected_* < 0.05). Section numbers refer to Montreal Neurological Institute coordinates. Abbreviations: HP = hippocampus, V_T_ = total distribution volume, WM = white matter.

When correcting for binding-affinity by normalizing VOI values to the cerebellum, group differences were found in the parietal lobe (*F* = 4.3, *p* = 0.020), with C+ patients showing higher V_T-ratio_ than HC (Figure 2A). With PVC, differences were found in the frontal, parietal, and occipital lobe (*F* ≥ 4.0, *p* ≤ 0.026), while C+ patients presented with higher V_T-ratio_ than HC and C− patients. When comparing V_T-ratios_ on a voxel-level, C+ patients presented with a higher V_T-ratio_ in the left occipital- (8.9 ± 6.9%, mean ± SD cluster increase) and right parietal lobe (9.0 ± 7.0%) when compared to HC and right parietal lobe when compared to C− patients (11 ± 9.9%). After PVC, an increased V_T-ratio_ was found in C+ patients in the left (9.2 ± 7.3%) and right occipital lobe (9.0 ± 6.5%), only compared to HC (all clusters *p_FWE_* < 0.05)(Figure 2B; Supplementary Table S3). No differences in VOI/voxel V_T-ratio_ between C− patients and HC were found. 

Fixel-based analysis of diffusion-weighted images revealed no group differences of WM-metrics (FD, log-FC, or FDC).

### 3.3. Clinical Parameters

Group differences were found for self-reported depression, anxiety, stress, fatigue, and cognitive complaints, with C+ patients consistently reporting higher scores than HC and higher depression levels than C− patients (Table 2 and Supplementary Table S4). C− patients also scored significantly higher than HC on the same scales, except for anxiety. Four C+ patients (22%) and one C− patient (6%) reported severe cognitive complaints [48]. Patient groups did not differ in GDS, but, when classifying patients as cognitively impaired, three C+ patients were classified as impaired (17%), while none of the C− patients received this classification. 

**Figure 3:**
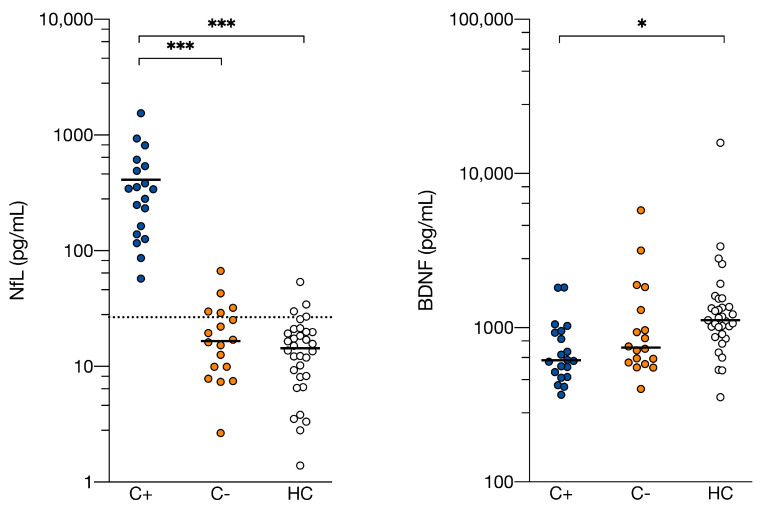
**Serum neurofilament light chain and plasma brain-derived neurotrophic factor levels are altered after chemotherapy treatment.** Neurofilament light chain (NfL) and brain derived-neurotrophic factor (BDNF) levels were measured in serum and plasma, respectively, of 19 C+ (breast cancer patients treated with chemotherapy), 18 C− (chemotherapy-naïve breast cancer patients) and 33 HC (healthy women) and showed to be altered in C+ compared to both control groups for NfL and only compared to HC for BDNF. Individual values with group medians are presented. The dotted line indicates diagnostic cutoff level for neurodegenerative disease with NfL immunoassay serum measurement. Group differences were assessed with ANOVA analysis with group as factor and age as covariate, with post-hoc least significance difference tests to assess which groups differ. *** *p* < 0.001, * *p* < 0.01.

Inflammatory (SM) and both neuronal (NfL, BDNF) blood markers differed between groups (Table 2, Figure 3). All 19 C+ patients (100%), five C− patients (28%), and four HC (11%) presented above a neurodegenerative NfL cut-off level [50]. C+ patients presented with 20-fold higher NfL (median 339 pg/mL, range 57–1543) compared to C− patients (17 pg/mL, range 3–67) and HC (14 pg/mL, range 1–54) (*F* = 105, *p* = 4.2E-21). Plasma levels of BDNF differed between groups (*F* = 4.3, *p* = 0.017), with C+ patients presenting lower levels than HC. No differences were found between C− patients and HC.

### 3.4. Associations for C+ Patients

**Figure 4:**
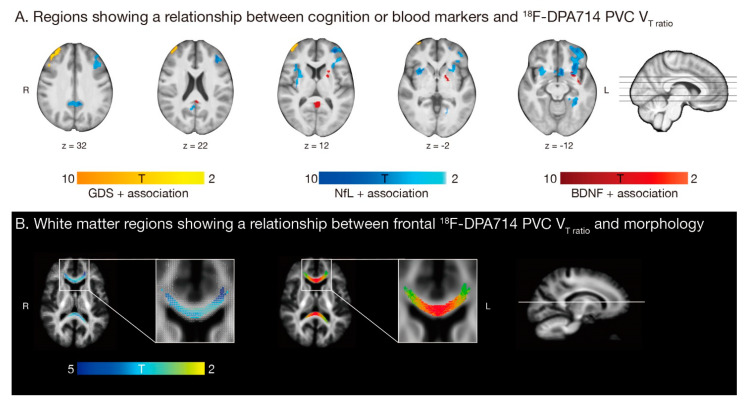
**The relationship between neuroinflammation and clinical parameters and white matter microstructure in 15 chemotherapy treated (C+) breast cancer patients.** (**A**) Global deficit score (GDS, orange; corrected for yes/no higher education), neurofilament light chain (NfL, blue; corrected for age) and brain-derived neurotrophic factor (BDNF, red; corrected for age) showed a significant positive association with partial-volume corrected (PVC) total distribution volume (V_T_)_-ratio_ in the temporal lobe, putamen, and caudate, and additionally in the frontal pole and insular cortex for NfL and parietal lobe for BDNF. (**B**) Fixels with a significant negative association with frontal V_T-ratio_ are shown on the left (blue-green, white = all fixels; corrected for age and intracranial volume). The right image shows streamlines passing through fixels showing a significant negative association (colored by direction; red: left-right, green: anterior-posterior, blue: inferior-superior), in the forceps major and minor of the corpus callosum. All models are with threshold *p_uncorrected_* < 0.001, *p_FWE-corrected_* < 0.05.

Whole brain voxel-wise regression analysis showed a higher GDS, but not CFQ score, associated with a higher V_T-ratio_ in the frontal lobe. Higher blood levels of NfL and BDNF, but not the inflammatory component (SM), were associated with higher V_T-ratio_ in the temporal lobe, putamen, and caudate, and additionally in the frontal pole and insular cortex for NfL and parietal lobe for BDNF. Secondly, whole brain fixel-based regression analysis showed higher frontal V_T-ratio_ was negatively associated with WM log-FC, but not FDC or FD, in the forceps-minor and -major of the corpus callosum (Figure 4).

## 4. Discussion

To our knowledge, this is the first in vivo study investigating the neuroinflammatory effect of chemotherapy and its relationship with cognition. When compared to chemotherapy-naïve or healthy women, increased neuroinflammation, associated with worse cognitive performance, and 20-fold higher levels of the axonal damage marker NfL were found in chemotherapy-treated patients with breast cancer. 

Increased relative glial expression, measured by TSPO-PET, was observed in chemotherapy-treated patients when compared to chemotherapy-naïve or healthy women, while no absolute differences were observed. Activation of glial cells leads to an ongoing pathologic process that includes neuroinflammation and cellular destruction. Since TSPO is equally expressed across different glia phenotypes, it is impossible to differentiate protective from destructive effects. However, previous tumor-bearing rodent models show chemotherapeutics trigger microglia activation [16], suggesting a neuroinflammatory reaction to various insults is present in these chemotherapy-treated patients. Alternatively, since clinical characteristics inherently differ between patients receiving chemotherapy or not, advanced disease status could possibly contribute to the observed neuroinflammatory effect.

Chemotherapy-treated patients presented with differential expression in several blood markers. Higher peripheral inflammation was found compared to healthy women, in concordance with literature [53,54]. This was also present in chemotherapy-naïve patients, as cancer can induce peripheral inflammation [8]. All nineteen chemotherapy-treated patients (100%) had levels of NfL, a neuronal survival marker, above a cut-off for neurodegenerative disease [55]. This was only the case for a subset of controls, corresponding to earlier observed distributions [50]. These results suggest chemotherapy-treatment induces axonal damage and/or neuronal degeneration. Peripheral neuropathy, a known side-effect of chemotherapy [56], could partially explain elevated NfL-levels, with earlier studies observing 2 to 7-fold increases [57,58]. However, central neurotoxicity, after which NfL is released into cerebrospinal fluid (CSF) and blood, could potentially explain the observed 20-fold higher concentrations. The remarkable long half-life of NfL (from weeks to months [59]) underscores the possibility of this being a chronic process. Interestingly, a recent study examining > 2000 individuals, found NfL blood levels were higher across all cortical neurodegenerative disorders, amyotrophic lateral sclerosis, and parkinsonian disorders, when compared to cognitively unimpaired controls, proposing age-related cut-offs to improve diagnosis [23]. This emphasizes the potential of NfL as a quick and accessible biomarker to indicate neurodegeneration in people who experience cognitive problems. Additionally, chemotherapy-treated patients showed lower BDNF levels, a neurotrophic growth-factor involved in brain plasticity. Lower blood BDNF is seen in neurodegenerative diseases, together with upregulation of pro-inflammatory cytokines in the brain, eventually causing neuronal death [60]. The inflammatory state seen after chemotherapy could potentially explain lower BDNF levels. In conclusion, while several blood markers could differentiate healthy women from patients treated for breast cancer, NfL emerged as the most sensitive for identifying chemotherapy-treated patients.

When evaluating cognition, deviation from the healthy subject’s norm was observed in 17% of chemotherapy-treated patients for objective and 22% for self-report measurements. This is in concordance with literature, showing that a subset of chemotherapy-treated breast cancer patients is more vulnerable to develop cognitive impairments [6,61]. Although research has primarily focused on breast cancer, cognitive change is observed across a variety of cancer types, each with their specific treatment protocol [6]. Rodent studies have found virtually all categories of cytotoxic agents can disrupt various neurobiological processes and induce cognitive impairment [62]. Further research will be necessary to study the neurotoxic effects of various drugs [63], to validate findings from this study in other cancer populations. Additionally, both chemotherapy-treated and chemotherapy-naïve patients scored higher on depression, stress, and fatigue scales than healthy women. The psychosocial impact of a cancer diagnosis and treatment should therefore not be underestimated.

A higher relative glial expression in chemotherapy-treated patients was associated with worse cognitive performance. While neuroinflammation has been widely speculated as a potential mechanism of CRCI [16,64], this is the first study to directly observe a relationship in a clinical sample. Psychosocial risk factors are unlikely to alter microglial activation in humans [65], suggesting that chemotherapy and its neurotoxic sequelae, rather than a chronic reduced mood-state, influence frontal glial overexpression.

Earlier diffusion-imaging studies have reported microstructural changes in the corpus callosum after chemotherapy [27,66,67]. This commissural fiber-bundle is especially vulnerable to demyelination and inflammation, potentially caused by its dense axonal packing and high vascular supply [68]. Using fixel-based analysis, we showed that a reduction in corpus callosum fiber cross-section was associated with frontal glial overexpression. This reduction is likely to reflect impaired ability to transfer information across brain regions, potentially indicating impaired WM integrity [37] and thereby possibly contributing to cognitive impairments seen in chemotherapy-treated patients.

Peripheral inflammation is known to associate with cognitive impairment [11,56]. While chemotherapy-treatment was associated with higher peripheral inflammation, no correlation was found between central and peripheral inflammation. This is in line with a recent study not observing differences in brain TSPO binding (using another radioligand) after an immune challenge [69]. The used TSPO target and quantification methods or the inflammatory composite component could be too insensitive to show subtle associations. Alternatively, the relationship is more complex or there simply is none. 

Remarkably, a positive association between BDNF and local glial hyperactivation was found. Because of its known neuroprotective role, a local acute proinflammatory state could provide a possible explanation, as BDNF is known to be directly involved in neuroinflammation activation [60]. However, rodent models show chronic treatment with chemotherapy, changes neuronal structure and reduces neurogenesis [14,70], suggesting a rather local protective effect. A positive relationship between NfL blood concentration and relative glial activation in other brain regions was found, indicating an association between neuroinflammatory and neurotoxic processes. Unregulated/chronic glial activation can lead to tissue destruction [19]. The glial–NfL association could therefore indicate a shift to a more chronic, harmful environment in the brain. Early on in multiple-sclerosis patients, a recent study found a positive association between a microglia-related protein and NfL in CSF [71], supporting the role of NfL as a potential biomarker for neuroinflammatory activity. Whether the relationships seen are neuroprotective or -destructive, cannot be disentangled by our study. 

This study has some limitations. First, calculating V_T-ratio_ reduces variability between subjects and makes a binding affinity covariate redundant, increasing sensitivity in analyses. However, measurements could be affected by alterations in the cerebellum, which can only serve as a pseudo-reference region. Nonetheless, cerebellar V_T_-values were the lowest of all cortical VOIs, did not differ between groups, and no GM atrophy was found in the cerebellum. A larger proportion than expected, based on previous mixed-gender studies, was classified as LA (30%), indicating women could be more prone to inherit the low-affinity polymorphism. Additionally, TSPO-signal can be driven by other factors than microglia activation, such as expression on astrocytes/endothelial cells (~25%), recruitment of monocytes into the parenchyma, and changes in BBB permeability [72,73]. Full kinetic modelling (as applied here) and correction for the endothelial component (SM) are proposed to partially account for such effects, but interpretation still warrants caution regarding the cellular specificity. Further studies using only HA, third generation TSPO-radioligands less sensitive to the binding polymorphism or more specific for microglial activation, will be necessary and could be more sensitive to detect absolute differences. 

Secondly, the observed differences cannot solely be attributed to chemotherapy, as cancer treatment entails a psychologically challenging trajectory [74]. Higher levels of stress, anxiety, depression, and fatigue were also observed in chemotherapy-treated patients. Moreover, clinical characteristics (e.g., cancer subtype/menopausal status) inherently differ between patients receiving chemotherapy or not, possibly contributing to the observed effects. For instance, hormonal differences are known to influence cognitive complaints [75]. Additionally, since the used treatment regime consisted of a combination of cytotoxic agents, this study cannot disentangle individual toxicities of the used agents. Lastly, we recognize that this study is limited by a relatively small cohort size. This potentially explains why no WM-microstructure group differences were detected. Larger longitudinal studies will be necessary to validate these findings, as well as to further elucidate influences of possible confounders and individual (or other) cytostatic agents, on neuro-inflammation and/or cognitive impairment.

## 5. Conclusions

This study showed that chemotherapy-induced neuroinflammation can be detected in vivo and is associated with worse cognitive functioning, higher levels of markers for neurotoxicity and -plasticity, and differences in WM-microstructure. Future studies are necessary to differentiate between underlying neuroprotective or -destructive mechanisms of the increased expression of inflammatory cells in the brain. Additionally, serum NfL could be an easily accessible biomarker to assess the toxic effects of chemotherapy and evaluate future neuroprotective therapeutic strategies.

## Figures and Tables

**Table 1:** Demographics and characteristics of the study population.

Characteristic	Whole Sample							PET Subsample						
	C+ *n* = 19		C− *n* = 18		HC *n* = 37		Group Difference *p* Value *	C+ *n* = 15		C− *n* = 15		HC *n* = 15		Group Difference *p* Value *
Age in years, mean (SD)	47	(10)	50	(6)	45	(10)	0.092	51	(8)	49	(6)	44	(10)	0.059
Education in years, mean (SD)	13	(4)	14	(3)	15	(2)	0.315	13	(3)	14	(3)	14	(2)	0.872
Body-mass index in kg/m^2^, mean (SD)	25	(4)	25	(5)	24	(3)	0.184	25	(4)	25	(5)	23	(3)	0.400
Postmenopausal at diagnosis, no. (%)	9	(47)	8	(44)	-		0.999 ^†^	9	(60)	6	(40)	-		0.273 ^†^
Post- or perimenopausal at assessment, no. (%)	15	(79)	8	(44)	13	(34)	0.006 ^†^	14	(93)	6	(40)	4	(27)	0.001 ^†^
Days since end of chemotherapy/surgery, mean (SD)	26	(17)	36	(12)	-		0.025	28	(13)	36	(12)	-		0.106
Breast cancer stage, no. (%)							<0.001 ^†^							0.002 ^†^
0–1	0	(0)	10	(55)	-		-	0	(0)	8	(53)	-		-
2	6	(32)	7	(39)	-		-	6	(40)	6	(40)	-		-
3	13	(68)	1	(6)	-		-	9	(60)	1	(1)	-		-
Cancer treatment, no. (%)														
Neo-adjuvant chemotherapy (EC + T)	8	(42)	-		-		-	8	(53)	-		-		-
Scheduled for radiotherapy	13	(68)	11	(61)	-		-	10	(67)	9	(60)	-		-
Scheduled for anti-hormone therapy	9	(47)	14	(78)	-		-	8	(53)	12	(80)	-		-
High affinity binders, no. (%)	-		-		-		-	9	(60)	7	(53)	7	(47)	0.809 ^†^
Injected activity in MBq, mean (SD)	-		-		-		-	144	(12)	144	(15)	144	(21)	0.983

* Group differences tested with ANOVA for continuous variables and chi-square tests for categorical variables (last indicated by ^†^). Abbreviations: C− = chemotherapy-naïve breast cancer patients, C+ = breast cancer patients treated with chemotherapy, HC = healthy controls, EC + T = 4 rounds of epirubicin 90 mg/m^2^ + cyclophosphamide 600 mg/m^2^ and 4–12 rounds of paclitaxel 80 mg/m^2^, SD = standard deviation.

**Table 2:** Neuropsychological assessment, questionnaires and blood markers.

	C+ *n* = 19		C− *n* = 18		HC *n* = 33		Group Difference *p* Value *	Post-hoc *p* Value		
								C+ vs. HC	C− vs. HC	C+ vs. C−
Cognition (C+ n = 18)										
Global deficit score, mean (SD)	0.27	(0.27)	0.21	(0.14)		-	0.388	-	-	-
Impaired, no. (%) (GDS)	3	(17)	0	(0)		-	0.286 ^†^	-	-	-
Self-report cognitively impaired, no. (%) (CFQ_TOT_)	4	(22)	1	(6)	0	(0)	0.434 ^†^	-	-	-
Self-report, mean (SD) (C+ *n* = 18)										
Beck depression inventory	10.83	(5.25)	7.28	(6.62)	4.11	(3.45)	<0.001	<0.001	0.040	0.034
Spielberger state-trait anxiety inventory	41.00	(11.62)	37.83	(14.36)	31.74	(9.09)	0.008	0.005	0.053	0.407
Self-perceived stress scale	16.67	(6.75)	15.44	(9.82)	9.20	(6.06)	0.001	0.001	0.006	0.638
Fatigue assessment scale	28.17	(6.41)	26.17	(7.39)	19.43	(5.79)	<0.001	<0.001	0.001	0.324
CFQ total score	39.61	(15.31)	33.50	(13.54)	25.38	(8.00)	0.001	<0.001	0.026	0.130
Inflammatory markers, median pg/mL (IQR)										
bNGF	18.20	(8.87)	16.52	(9.80)	19.29	(9.48)	0.961	-	-	-
CRP, mg/L	1.20	(0.90)	1.30	(2.75)	0.85	(0.65)	0.240	-	-	-
Eotaxin	27.07	(5.85)	29.58	(11.84)	28.66	(12.31)	0.555	-	-	-
IFN-g	3.39	(1.91)	3.39	(1.55)	2.68	(1.27)	0.471	-	-	-
IL-1a	1.20	(0.93)	1.29	(1.72)	1.03	(1.01)	0.668 ^†^	-	-	-
IL-1b	1.16	(0.82)	2.27	(3.00)	1.16	(1.23)	0.440 ^†^	-	-	-
IL-4	12.70	(5.54)	11.87	(7.10)	10.50	(3.88)	0.578	-	-	-
IL-6	7.66	(5.04)	6.41	(3.02)	4.60	(2.98)	0.031	0.010	0.402	0.093
IL-8	9.68	(3.81)	8.58	(12.23)	5.31	(3.81)	0.001	0.008	0.001	0.446
IL-10	1.24	(0.93)	0.97	(0.63)	0.97	(0.66)	0.638	-	-	-
IL-12	2.00	(0.40)	2.10	(0.35)	1.95	(0.27)	0.187	-	-	-
IL-18	36.55	(27.75)	31.10	(7.85)	33.47	(24.51)	0.751	-	-	-
MCP-1	70.33	(33.35)	61.54	(25.56)	47.64	(16.70)	<0.001	<0.001	0.033	0.069
MIP-1B	146.36	(101.06)	105.73	(121.97)	79.78	(53.70)	0.006	0.002	0.098	0.210
TNF-a	0.64	(1.24)	1.39	(2.44)	0.88	(1.39)	0.575 ^†^	-	-	-
VCAM-1	93294	(41634)	73552	(47979)	91143	(52101)	0.051	-	-	-
VEGF-A	24.15	(18.88)	27.56	(15.50)	31.98	(24.97)	0.338	-	-	-
Neuronal survival markers, median pg/mL (IQR)										
BDNF	617.24	(472.04)	741.98	(843.38)	1110.17	(482.29)	0.010	0.011	0.484	0.089
NfL	339.00	(399.00)	16.55	(19.84)	14.38	(11.68)	<0.001	<0.001	0.475	<0.001

* Group differences tested with ANOVA for continuous variables, with years of education (GDS) or age (blood markers) as covariate, and chi-square tests for categorical variables (last indicated by ^†^). When no main group effect was found, subsequent least-square difference tests were not performed (-). All blood biomarkers were log-transformed for analysis. 50% of Il-1a, IL-1b and TNF-a levels were very close to the detection limit and therefore converted to a 1 (equal or above detection limit) or 0 (below detection limit) variable. Abbreviations: BDNF = brain-derived neurotrophic factor, bNGF = beta nerve growth factor, CFQ = cognitive failure questionnaire, CRP= C-reactive protein, C− = chemotherapy-naïve breast cancer patients, C+ = breast cancer patients treated with chemotherapy, GDS = global deficit score, HC = healthy controls, IFN-g = interferon gamma, IL = interleukin, IQR = inter-quartile range, MCP-1 = monocyte chemoattractant protein 1, MIP-1 = macrophage inflammatory protein 1, NfL = neurofilament light-chain, TNF-a = tumor necrosis factor alpha, VCAM-1 = vascular cell adhesion molecule 1, VEGF-A = vascular endothelial growth factor.

## Data Availability

The data that support the findings of this study are available on request from the corresponding author.

## Supplementary Materials

The following are available online at https://www.mdpi.com/article/10.3390/cancers13164198/s1, Table S1: Primer characteristics for Next Generation Sequencing, Table S2: LGA [^18^F]DPA714 V_T_ for cortical and WM volumes of interest, Table S3: Clusters showing significant differences for neuroimaging modalities, Table S4: Overview of neuropsychological measures, Table S5: First component of principal component analysis on four blood inflammatory markers, Figure S1: Average parametric LGA V_T-ratio_ images for 15 C+ patients, 15 C− patients and 15 HC, Figure S2: Regions showing [^18^F]DPA714 V_T-ratio_ differences with 2TCM + vascular trapping, Supplementary materials and methods, Supplementary Results.
