# Correction: Schroyen et al. Neuroinflammation and Its Association with Cognition, Neuronal Markers and Peripheral Inflammation after Chemotherapy for Breast Cancer. *Cancers* 2021, *13*, 4198

**DOI:** 10.3390/cancers15123091

**Published:** 2023-06-07

**Authors:** Gwen Schroyen, Jeroen Blommaert, Donatienne van Weehaeghe, Charlotte Sleurs, Mathieu Vandenbulcke, Nina Dedoncker, Sigrid Hatse, An Goris, Michel Koole, Ann Smeets, Koen van Laere, Stefan Sunaert, Sabine Deprez

**Affiliations:** 1 Leuven Brain Institute, KU Leuven, 3000 Leuven, Belgium; jeroen.blommaert@kuleuven.be (J.B.); charlotte.sleurs@kuleuven.be (C.S.); mathieu.vandenbulcke@kuleuven.be (M.V.); nina.dedoncker@kuleuven.be (N.D.); an.goris@kuleuven.be (A.G.); koen.vanlaere@kuleuven.be (K.v.L.); stefan.sunaert@kuleuven.be (S.S.); sabine.deprez@kuleuven.be (S.D.); 2 Leuven Cancer Institute, KU Leuven, 3000 Leuven, Belgiummichel.koole@kuleuven.be (M.K.); ann.smeets@kuleuven.be (A.S.); 3 Department of Imaging and Pathology, KU Leuven, 3000 Leuven, Belgium; 4 Department of Oncology, KU Leuven, 3000 Leuven, Belgium; 5 Department of Nuclear Medicine and Molecular Imaging, KU Leuven, 3000 Leuven, Belgium; donatienne.vanweehaeghe@kuleuven.be; 6 Nuclear Medicine and Molecular Imaging, University Hospitals Leuven, 3000 Leuven, Belgium; 7 Department of Neurosciences, KU Leuven, 3000 Leuven, Belgium; 8 Psychiatry, University Hospitals Leuven, 3000 Leuven, Belgium; 9 Surgical Oncology, University Hospitals Leuven, 3000 Leuven, Belgium; 10 Radiology, University Hospitals Leuven, 3000 Leuven, Belgium

In the original publication [1], there was a mistake in Figure 2A as published. The upper panel was incorrectly a duplicate version of the lower panel, and included PVC V_T_ values, instead of uncorrected V_T_ values. In the caption, a typo in referring to legend colors (blue instead of red image color) was also observed. The corrected Figure 2 appears below. The accompanying text in the manuscript does not include these mistakes and therefore remains unchanged. The authors state that the scientific conclusions are unaffected. This correction was approved by the Academic Editor. The original publication has also been updated.

## Figures and Tables

**Figure 2:**
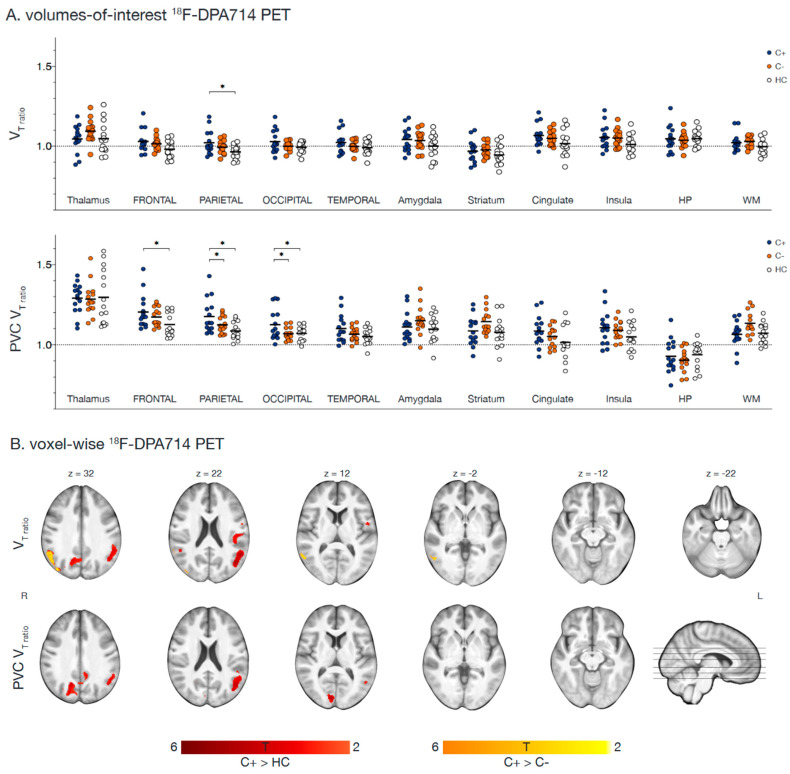
**Regions showing [^18^F]DPA714 V_T-ratio_ differences with LGA.** Fifteen chemotherapy-treated patients (C+) were assessed for [^18^F]DPA714 V_T-ratio_ and compared to 15 chemotherapy-naïve patients (C−) and 15 healthy women (HC). (**A**) Volumes-of-interest based logan-graphical analysis (LGA) results of 11 volumes of interest are presented, showing higher V_T-ratio_ in C+ patients compared to HC in the parietal lobe. After partial volume correction (PVC), C+ patients showed higher V_T-ratio_ compared to C− and HC in the parietal and occipital lobe and additionally in the frontal lobe when compared to HC (* *p* < 0.05). (**B**) Voxel-based whole brain LGA results are presented, showing higher V_T-ratio_ in C+ patients compared to HC (red) and C− patients (orange) in the occipital and parietal lobe. After PVC, only differences between C+ and HC persisted for V_T-ratio_ images (all *p_uncorrected_* < 0.005, *p_cluster FWE-corrected_* < 0.05). Section numbers refer to Montreal Neurological Institute coordinates. Abbreviations: HP = hippocampus, V_T_ = total distribution volume, WM = white matter.
