# Supplementary material for: Neuroinflammation and Its Association with Cognition, Neuronal Markers and Peripheral Inflammation after Chemotherapy for Breast Cancer
Source: Cancers (Basel). 2021 Aug 20;13(16):4198. doi: 10.3390/cancers13164198 (PMC8391457; doi:10.3390/cancers13164198)
Supplement: Supplementary file 1 [file cancers-13-04198-s001.zip › cancers-1279541-supplementary.pdf]

Supplementary

Materials and methods

Genotyping

The single-nucleotide polymorphism c.439A > G (rs6971, p.Thr147Ala) was analyzed by Next Generation Sequencing. Genomic DNA was extracted using Chemagic DNA blood special 4ml kit (Chemagen-Perkin Elmer, Baesweiler, Germany) on a Chemagen MSM automate (Chemagen-Perkin Elmer, Baesweiler, Germany). DNA purity was measured using a DropSense96 (Trinean, Belgium) with cDrop™ Software. Polymerase chain reaction (PCR) was carried out in a volume of 20 µl with approximately 200ng DNA and 0.250µM forward and reverse primer, using Phusion High-Fidelity PCR Master Mix with HF Buffer (Thermo Scientific™, US). PCR was performed using denaturation (120s, 98°C), 35 cycles of denaturation (30s, 98°C) and hybridization/elongation (30s, 72°C) and a final extension (5min, 72°C). Primer-dimers were removed using SPRI bead technology. The samples were pooled at a concentration of 2nM. Sequencing was performed using Illumina MiSeq, HiSeq2500 and HiSeq4000 platforms.

Supplementary Table S1. ***Primer characteristics for Next Generation Sequencing.***

Primer[1]	Illumina P5/P7 Seq	Barcode	Illumina R1/R2 Seq	Primer
Forward	AATGATACGGCGACCACCGAGATCTAC AC	TAGATCGC	ACACTCTTTCCCTACACGACGCTCT TCCGATCT	CTCTACCCCTACCT GGCCT
Reverse	CAAGCAGAAGACGGCATACGAGAT	TAGTATAG	GTGACTGGAGTTCAGACGTGTGCT CTTCCGATCT	GGCCACCACATCA CAAGC

After demultiplexing, reads were mapped to the human reference genome (hg19) using BWA mem (v0.7.17)[2]. The obtained BAM files were sorted and indexed using SAMtools[3] (v1.5) prior to variant calling with freebayes (v1.3.2-40-gcce27fc). Only reads mapped with a quality above or equal to 20 and bases with a quality above or equal to 10 were considered for variant calling[4].

Image acquisition and reconstruction

PET data were reconstructed (vendor MP26 software version) with ordered subset maximum likelihood expectation maximization with 4 iterations and 28 subsets followed by postfiltering with in-plane 4 mm gaussian post-smoothing. Attenuation correction was performed using a validated ZTE based approach[5]. Data were binned into 20 frames (4x15s, 4x60s, 2x150s and 10x300s; FOV 192x192x89; pixel size 1.56x1.56x2.78 mm^3) and rigid co-registration of every frame to the averaged first 10 frames was performed to correct for motion. During PET acquisition, 5mL arterial samples were manually collected from 10 seconds post injection until the end of the scan with gradual increasing time intervals. A total of 20 blood samples were centrifuged, and radioactivity in plasma samples was counted with a gamma counter, which was cross-calibrated with the PET-MR scanner. Additionally, at 5, 10, 20, 45- and 60-minutes post-injection, 5 mL arterial blood samples were collected to derive the parent free fraction.

Simultaneous with PET acquisition, a 3-dimensional volumetric sagittal T1-weighted image (3D BRAVO, TR/TE=8.5ms/3.2ms, FA=12°, 1.4×1×1 mm^3 voxel size, matrix 166×256×256), zero-echo-time MR for attenuation correction[5] (3D radial acquisition; FA=0.8°; 2.4×2.4×2.4 mm^3 voxel size, matrix 110×110×116, number of averages 4, bandwidth ± 62.5 kHz), multi-shell diffusion (b-values=0/700/1000/2000 with respectively 8/20/32/60 uniformly distributed gradient directions; TR/TE = 10335ms/86.3ms; FA=90°; 2.5x2.5x2.5 mm^3 voxel size; matrix 96x69x47, phase encoding=AP, with 8 b0 and 6 b2000 additional diffusion images with reversed phase-encoding) were acquired.

Image analysis

For quantification of [^{18}F]DPA714 PET, first, V_T voxel-based images were created using a plasma-input based logan graphical analysis, with an equilibration time (t*) of 36 minutes and blood volume fixed to 5% in PMOD (v3.8, PMOD technologies, Zurich, Switzerland). T1-weighted MRI were bias-field corrected, using ANTs N4 Bias-field correction[8]. Afterwards, the SPM12[9] CAT12[10] toolbox was used for image registration, segmentation, spatial normalization (DARTEL algorithm) and calculating modulated grey-matter (GM) volume maps. Region-based voxel-wise partial-volume correction[37] (PVC) was performed on V_T images with a 5.5mm isotropic full width at half maximum to mimic the PET image resolution, with PetSurfer and the Desikan-Killiany atlas for region delineation[6] (Freesurfer v6; Martinos Center for Biomedical Imaging, Harvard-MIT, Boston-USA). V_T and PVC V_T images were

spatially normalized, applying ANTs[7] normalization parameters from co-registered T1w MR images, to Montreal Neurological Institute space, and evaluated for group differences. Both T1w modulated GM and (PVC) V_T maps were smoothed with an isotropic gaussian kernel of 8-mm full width at half maximum. The spatially normalized and smoothed images were analyzed with SPM12[9]. Second, a volume-of-interest–based (VOI) analysis with RBV correction was conducted using the Hammers N30R83 maximum probability atlas to confirm findings at the voxel level in PMOD. A logan model and two-compartmental model with vascular trapping[11] were used to determine average PVC V_T in the 12 VOIs.

For WM micro- and macrostructure, the recommended pipeline for DWI pre-processing (https://mrtrix.readthedocs.io/en/0.3.16/workflows/DWI_preprocessing_for_quantitative_analysis.html, accessed March 2020) and fixel-based analysis using MRtrix[12] (v3.0) on diffusion-weighted images was followed (https://mrtrix.readthedocs.io/en/0.3.16/workflows/fixel_based_analysis.html, accessed March 2020)[13]. A fixel summarizes the mean direction and partial volume of multiple fiber bundles present within a voxel. This analysis included: motion-, distortion- and biasfield correction of raw diffusion images, estimating average group response functions (dhollander), estimation of individual Fibre Orientation Distribution (FOD) with Constrained Spherical Deconvolution (CSD), joint biasfield correction and intensity normalization, generating an FOD population template, generating a fixel mask, registering the individual FODs to the template, estimating fixels and assigning fixels to template fixels. Connectivity-based fixel enhancement[14] was implemented with 2 million streamlines. Fixels traversed by less than 150 streamlines were excluded from the final analysis (fixel mask).

Blood markers

Venous blood was collected in tubes (one 4mL EDTA and two 5 mL BCA) and centrifuged at 1200xg for 10 minutes. Supernatant (plasma and serum, respectively) was divided into aliquots and stored at -80 °C until analysis. Blood inflammatory markers and brain-derived-neurotrophic factor (BDNF) were determined by bead-based immunoassay with Aimplex (ImTec Diagnostics, Antwerp and YSL AimPlex, BioLegend, San Diego) 'premixed human inflammation 10-plex panel' [interferon-γ (IFNγ), Interleukin (IL) 1α (IL-1α), IL-1β, IL-6, IL-8, IL-10, IL-12p70, IL-18, monocyte chemoattractant protein 1 (MCP-1), and tumor necrosis factor α (TNFα)] and an Aimplex custom kit [BDNF, beta nerve growth factor (bNGF), Eotaxin, IL-4, vascular cell adhesion molecule 1 (VCAM-1), vascular endothelial growth factor

A (VEGF-A)] on plasma. CRP was determined by immunoturbidimetric assay (Gudnersen Health System) on plasma. All blood data were log10 transformed. Three cytokines with levels below or equal to the detection threshold in >50% of individuals (IL-1a, IL-1b and TNFα) deviated from a normal distribution even after log10 transformation and were hence transformed into a binary variable representing positive (above detection threshold) or negative (below or equal to detection threshold) measurements. NfL samples for which duplicate results were below the standard curve fit (N=2), were excluded from analysis.

Neuropsychological assessment

Cognitive functioning was assessed for the following domains: attention and concentration (WAIS digit span and letter number[15], Bourdon-Wiersma[16]), memory (Adult Verbal Learning[17]), executive functioning (Controlled Oral Word Association[18] and Stroop Color and Word[19]) and cognitive/psychomotor processing speed (WAIS digit symbol[15], Trail Making Test[18] and Nine Hole Peg test[20]). As described earlier[46], *T*-scores were calculated (based on the mean of the HC group), converted to a test-specific deficit score and averaged over 22 outcome measurements to derive a Global Deficit score (GDS). Participants completed the following questionnaires: Spielberger State-Trait Anxiety Inventory (STAI)[44], Beck Depression Inventory (BDI)[45], Perceived Stress Scale (PSS)[46], Fatigue Assessment Scale (FAS)[47] and the Cognitive Failure Questionnaire (CFQ)[48].

Results

[18F]DPA714 TSPO PET: neuroinflammation

Exploratively, when dividing groups based on binding affinity, only HA showed a difference in V_T in the occipital lobe (p=.04, F=2.7), with C+ patients presenting with higher V_T (3.2±0.9, mean ± SD) when compared to C- (2.2±0.5; p=.02) and HC (3.0±1.0, p=.04). Results were comparable when assessed with a two-compartment model with vascular trapping (Supplementary Figure 2).

Clinical parameters

NfL measurement showed a mean intra-plate variability (coefficient of variation) of 13% and a mean inter-plate variability of 28%. We checked consistency between standard curves across plates, with R^2 = 0.998 (*p*=1.2E-8). The majority of serum samples of the C+ group (n=17) and a subsample of the C- group (n=7) was re-analyzed one week after initial analysis, again in triplicate. Correlation between test-retest concentrations of the two mean measurements was very high: R^2 = .98, p < 2E-16.

Inflammatory (i.e. IL-6, IL-8, MCP-1, MIP-1b) blood markers differed between groups (Table2, Fig.3). Additionally, C+ patients presented with higher levels of IL-6, IL-8, MIP-1b and MCP-1 compared to HC (*F*>3.7, *p*<.031). C- patients also showed higher levels of IL-8 and MIP-1b than HC. No differences between C+ and C- patients were found.

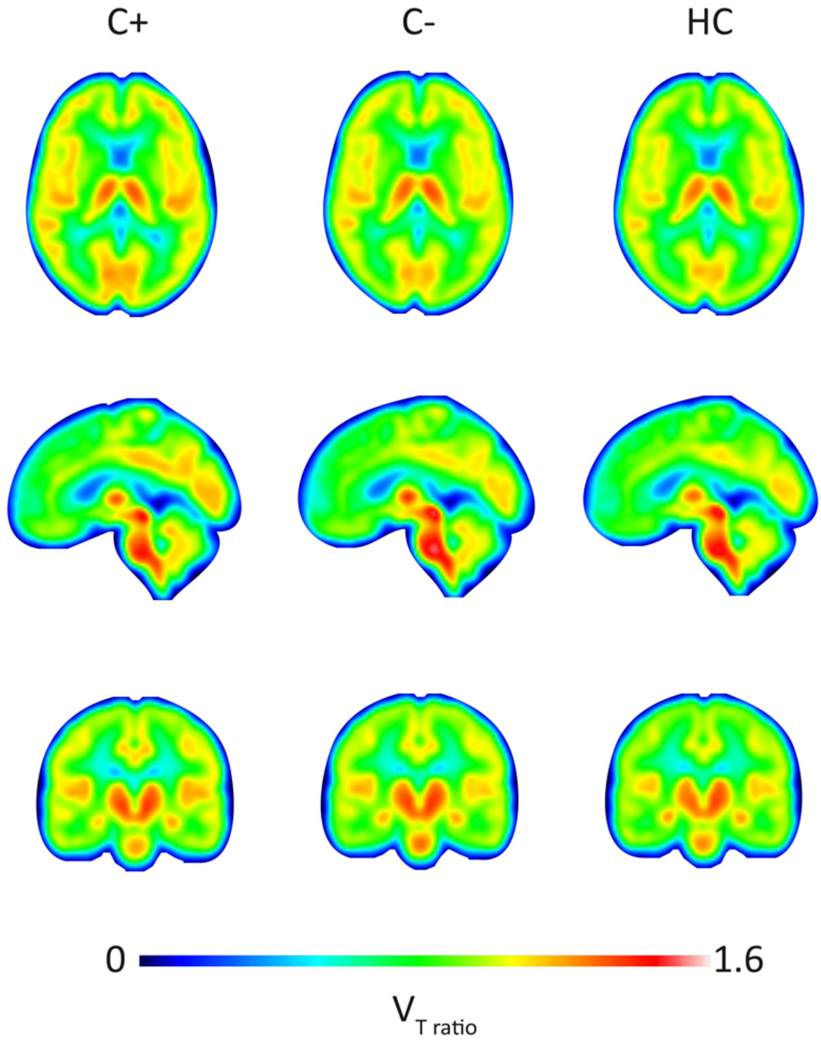

Supplementary Figure S1. ***Average parametric LGA $V_{T\ ratio}$ images for 15 C+ patients, 15 C- patients and 15 HC.*** *Abbreviations: C- = chemotherapy-naïve breast cancer patients, C+ = breast cancer patients treated with chemotherapy, HC = healthy controls, LGA = logan-graphical analysis, V_T = total distribution volume.*

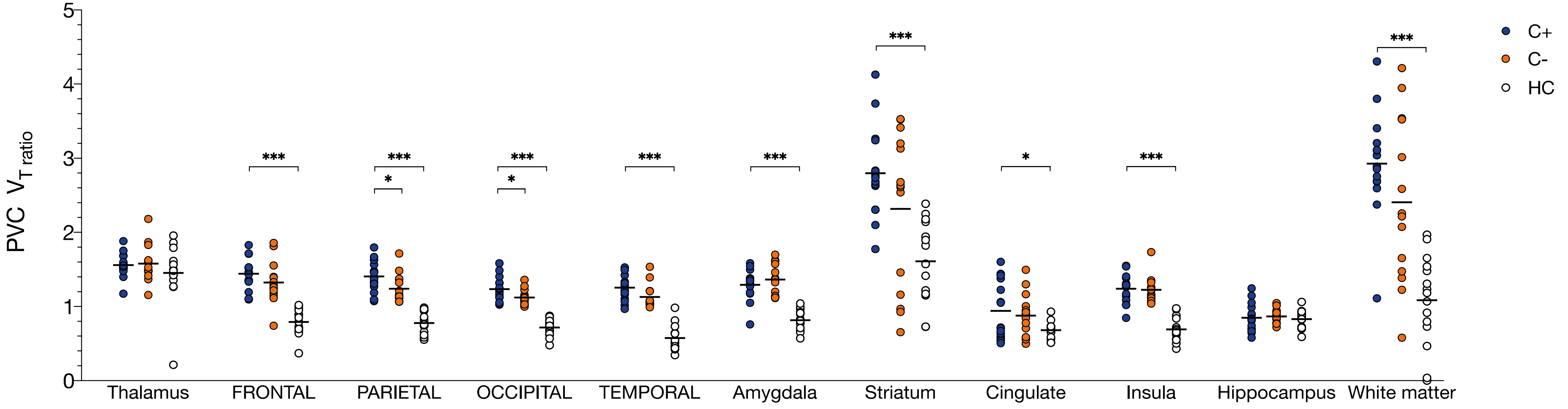

Supplementary Figure S2. ***Regions showing [^{18}F]DPA714 $V_{T\ ratio}$ differences with 2TCM + vascular trapping.*** *Fifteen chemotherapy-treated patients (C+) were assessed for differences in [^{18}F]DPA714 $V_{T\ ratio}$ and compared to 15 chemotherapy-naïve patients (C-) and 15 healthy women (HC). Volume-of-interest two-tissue compartment modelling with vascular trapping showing PVC $V_{T\ ratios}$ of 11 volumes of interest. C+ patients presented with relative higher PVC V_T in the parietal and occipital lobe when compared to both control groups, and additionally in the frontal lobe, temporal lobe, amygdala, striatum, cingulate, insula and white matter when compared to HC (*p<.05, ***p<.001).*

Abbreviations: PVC = partial volume correction, V_T =total distribution volume, 2TCM = two-tissue compartment model.

Supplementary Table S2. ***LGA [^{18}F]DPA714 V_T for cortical and WM volumes of interest***

Region<br>V_T (mean ± SD)	C+<br>n = 15	C-<br>n = 15	HC<br>n = 15
frontal cortex	1.99 ± 0.93	1.67 ± 0.45	1.77 ± 0.93
temporal cortex	1.97 ± 0.88	1.64 ± 0.43	1.77 ± 0.87
occipital cortex	1.98 ± 0.91	1.65 ± 0.43	1.78 ± 0.88
parietal cortex	1.97 ± 0.91	1.64 ± 0.43	1.74 ± 0.90
insular cortex	2.05 ± 0.99	1.74 ± 0.48	1.84 ± 0.99
cingulate cortex	2.07 ± 0.99	1.73 ± 0.47	1.86 ± 1.05
cerebellum	1.93 ± 0.92	1.65 ± 0.45	1.79 ± 0.90
white matter	1.84 ± 0.79	1.62 ± 0.44	1.69 ± 0.82

Fifteen chemotherapy-treated patients (C+) were assessed for [^{18}F]DPA714 V_T and compared to 15 chemotherapy-naïve patients (C-) and 15 healthy women (HC). Abbreviations: LGA = Logan graphical analysis, SD = standard deviation, V_T =total distribution volume, WM = white matter

Supplementary Table S3. ***Clusters showing significant differences for neuroimaging modalities.***

k_E	$p_{cluster}$ FWE-corrected	p_{peak} uncorrected	T	peak coördinates (mm) x	y	z	peak region (Harvard-Oxford (sub)cortical atlas / JHU WM atlas)	H	Contrast	Outcome (modality)	Covariates
Group comparison with C+, C- and HC											
1235	.008	<.001	5.40	-46	-64	18	lateral occipital cortex (occipital lobe)	L	C+ > HC	$V_{T\ ratio}$ (^{18}F-DPA-PET)	-
900	.048	.002	4.37	50	-46	24	angular gyrus (parietal lobe)	R	C+ > HC	$V_{T\ ratio}$ (^{18}F-DPA-PET)	-
1066	.017	<.001	4.17	56	-52	34	angular gyrus (parietal lobe)	R	C+ > C-	$V_{T\ ratio}$ (^{18}F-DPA-PET)	-
872	.013	<.001	4.69	-46	-66	18	lateral occipital cortex (occipital lobe)	L	C+ > HC	PVC $V_{T\ ratio}$ (^{18}F-DPA-PET)	-
769	.023	.001	4.14	10	-86	10	intracalcerine cortex (occipital lobe)	R	C+ > HC	PVC $V_{T\ ratio}$ (^{18}F-DPA-PET)	-
Regression analysis in C+ patients											
971	.005	<.001	7.37	40	52	18	frontal pole (frontal lobe)	R	positive association with GDS	PVC $V_{T\ ratio}$ (^{18}F-DPA-PET)	education
2125	<.001	<.001	10.49	-34	50	10	frontal pole (frontal lobe)	L	positive association with NfL	PVC $V_{T\ ratio}$ (^{18}F-DPA-PET)	age
433	.001	<.001	9.97	-36	-36	-20	temporal fusiform gyrus (temporal lobe)	L	positive association with NfL	PVC $V_{T\ ratio}$ (^{18}F-DPA-PET)	age
791	<.001	<.001	8.64	26	14	4	putamen (basal ganglia)	R	positive association with NfL	PVC $V_{T\ ratio}$ (^{18}F-DPA-PET)	age
344	.003	<.001	8.56	-6	-46	30	cingulate gyrus (parietal lobe)	Both	positive association with NfL	PVC $V_{T\ ratio}$ (^{18}F-DPA-PET)	age
294	.007	<.001	8.35	-8	8	-14	accumbens (basal ganglia)	Both	positive association with NfL	PVC $V_{T\ ratio}$ (^{18}F-DPA-PET)	age
271	.015	.001	9.90	-34	2	-18	temporal pole (temporal lobe)	L	positive association with BDNF	PVC $V_{T\ ratio}$ (^{18}F-DPA-PET)	age
281	.013	.001	4.83	36	10	-32	temporal pole (temporal lobe)	R	positive association with BDNF	PVC $V_{T\ ratio}$ (^{18}F-DPA-PET)	age
214	.042	.003	4.23	-18	4	4	pallidum/ putamen (basal ganglia)	L	positive association with BDNF	PVC $V_{T\ ratio}$ (^{18}F-DPA-PET)	age
249	.022	.001	4.18	-2	-46	14	posterior cingulate gyrus (parietal lobe)	Both	positive association with BDNF	PVC $V_{T\ ratio}$ (^{18}F-DPA-PET)	age
851	.017	<.001	4.97	-1	23	9	forceps minor corpus callosum (WM)	Both	negative association with frontal PVC $V_{T\ ratio}$	FBA log FC (DWI) *	age, ICV

** results are given for fixels, instead of voxels.*

Abbreviations: BDNF = brain-derived neurotrophic factor, C- = chemotherapy-naïve breast cancer patients, C+ = breast cancer patients treated with chemotherapy, DWI = diffusion weighted imaging, FBA = fixel-based analysis, GDS = global deficit score, H = hemisphere, HC = healthy controls, ICV = intracranial volume, NfL = neurofilament light chain, PVC = partial volume correction, V_T = distribution volume, WM = white matter.

Supplementary Table S4. *Overview of neuropsychological measures*

Domain<br>test, mean score (SD)	C+<br>n = 18	C-<br>n = 18	HC<br>n = 34	Group comparison<br>*p* value	Post-hoc *p* value<br>C+ vs HC	 C- vs HC	 C+ vs C-
Attention and concentration							
Bourdon-Wiersma, avg/row (s)	11.71 (1.52)	12.01 (2.37)	11.17 (2.38)	.433	-	-	-
Bourdon-Wiersma, SD	1.25 (0.50)	1.31 (0.45)	1.18 (0.45)	.079	-	-	-
Bourdon-Wiersma, omissions	10.88 (12.65)	7.57 (6.01)	10.63 (6.07)	.398	-	-	-
WAIS backward digit span, max	4.94 (1.11)	5.56 (2.04)	4.65 (1.54)	.166	-	-	-
WAIS backward digit span, total	8.56 (1.79)	8.11 (2.03)	7.94 (2.31)	.405	-	-	-
WAIS forward digit span, max	5.44 (1.29)	5.33 (1.41)	5.82 (1.03)	.450	-	-	-
WAIS forward digit span, total	7.89 (2.30)	7.33 (2.89)	8.79 (1.77)	.142	-	-	-
WAIS letter number, max	5.39 (1.15)	5.50 (1.04)	5.24 (1.23)	.339	-	-	-
WAIS letter number, total	18.06 (4.19)	19.06 (3.23)	18.85 (3.46)	.735	-	-	-
Executive functioning							
Controlled oral word association (BDH)	38.61 (11.16)	40.17 (14.34)	38.56 (8.87)	.663	-	-	-
Stroop color word, card A (s) #	46.89 (7.93)	45.28 (5.44)	42.79 (6.90)	.167	-	-	-
Stroop color word, card B (s) #	57.06 (9.76)	56.72 (6.68)	53.06 (8.57)	.249	-	-	-
Stroop color word, card C (s) #	86.44 (17.10)	87.56 (11.54)	83.15 (87.56)	.856	-	-	-
Stroop color word, interference (s) #	34.47 (12.79)	36.56 (10.27)	34.21 (10.27)	.929	-	-	-
Memory							
AVLT -learning (sum A1-A5)	54.28 (8.18)	58.22 (6.80)	56.21 (7.33)	.296	-	-	-
AVLT -recall (A7)	11.17 (3.05)	12.83 (1.76)	11.06 (2.30)	.023	.649	.263	.045
AVLT -recognition	20.44 (4.67)	22.06 (5.08)	19.12 (5.24)	.073	-	-	-
Processing speed							
Trail making A (s) #	28.28 (9.47)	27.78 (9.25)	22.47 (6.23)	.021	.016	.027	.849
Trail making B (s) #	64.67 (26.27)	57.78 (18.44)	49.76 (21.94)	.143	-	-	-
WAIS digit symbol	78.67 (14.24)	76.56 (12.44)	84.35 (12.62)	.142	-	-	-
9HPT-dominant hand (s) #	68.94 (10.13)	62.72 (9.50)	58.24 (9.22)	.001	.001	.112	.055
9HPT-non dominant hand (s) #	77.39 (13.21)	65.11 (7.98)	62.71 (10.34)	<.001	<.001	.454	.001

Higher scores indicate better performance, except when indicated with #. ANCOVA model included group as factor and years of education as covariate. Abbreviations: AVLT = adult verbal learning test, AVLT -recognition = subtracted false alarms from correctly identified words in the memory trial, C- = chemotherapy-naïve breast cancer patients, C+ = breast cancer patients treated with chemotherapy, HC = healthy controls, 9HPT = nine-hole peg test, WAIS = Wechsler adult intelligence scale.

Supplementary Table S5. ***First component of principal component analysis on four blood inflammatory markers.***

Component	Loading	Communality / total variance
interleukin 8 (IL-8)	.788	.606
monocyte chemoattractant protein 1(MCP-1)	.770	.593
interleukin 6 (IL-6)	.618	.382
macrophage inflammatory protein (MIP-1)	.569	.323

With principal component analysis on the four blood inflammatory markers showing group differences, the first component was extracted, accounting for 47% of the variance and with an eigenvalue of 1.9 (Kaiser-Meyer-Olkin test value[21] = 0.679, Bartlett's test of Sphericity p =1.1E--5).

References

1. Lange, V. *et al.* Cost-efficient high-throughput HLA typing by MiSeq amplicon sequencing. *BMC Genomics* **15**, 63 (2014).

2. Li, H. & Durbin, R. Fast and accurate short read alignment with Burrows-Wheeler transform. *Bioinformatics* **25**, 1754–1760 (2009).

3. Li, H. *et al.* The Sequence Alignment/Map format and SAMtools. *Bioinformatics* **25**, 2078–2079 (2009).

4. Garrison, E. & Marth, G. Haplotype-based variant detection from short-read sequencing. (2012).

5. Schramm, G. *et al.* Regional Accuracy of ZTE-Based Attenuation Correction in Static [18F]FDG and Dynamic [18F]PE2I Brain PET/MR. *Front. Phys.* **7**, 211 (2019).

6. Greve, D. N. *et al.* Different partial volume correction methods lead to different conclusions: An 18F-FDG-PET study of aging. *Neuroimage* **132**, 334–343 (2016).

7. Avants, B. B. *et al.* A reproducible evaluation of ANTs similarity metric performance in brain image registration. *Neuroimage* **54**, 2033–2044 (2011).

8. Tustison, N. J. *et al.* N4ITK: Improved N3 Bias Correction. *IEEE Trans. Med. Imaging* **29**, 1310–1320 (2010).

9. Friston, K., Holmes, A. & Worsley, K. Statistical parametric maps in functional imaging: a general linear approach. *Hum. Brain Mapp.* (1994).

10. Gaser, C. & Dahnke, R. CAT - A Computational Anatomy Toolbox for the Analysis of Structural MRI Data. in *HBM Conference 2016* (2016).

11. Wimberley, C. *et al.* Impact of endothelial 18-kDa translocator protein on the quantification of 18 F-DPA-714. *J. Nucl. Med.* **59**, 307–314 (2018).

12. Tournier, J.-D. *et al.* MRtrix3: A fast, flexible and open software framework for medical image processing and visualisation. *bioRxiv* (2019). doi:10.1101/551739

13. Raffelt, D. A. *et al.* Investigating white matter fibre density and morphology using fixel-based analysis. *Neuroimage* **144**, 58–73 (2017).

14. Raffelt, D. A. *et al.* Connectivity-based fixel enhancement: Whole-brain statistical analysis of diffusion MRI measures in the presence of crossing fibres. *Neuroimage* **117**, 40–55 (2015).

15. Wechsler, D. Wechsler Adult Intelligence Scale—Fourth Edition. (2008).

16. Grewel, F. The Bourdon-Wiersma test. *Folia Psychiatr. Neurol. Neurochir. Neerl.* **56**, 694–703 (1953).

17. Rey, A. *L'examen clinique en psychologie. [The clinical examination in psychology].* (Presses Universitaries De France, 1958).

18. Carone, D. A. E. Strauss, E. M. S. Sherman, & O. Spreen, A Compendium of Neuropsychological Tests: Administration, Norms, and Commentary . *Appl. Neuropsychol.* **14**, 62–63 (2007).

19. Stroop, J. R. Studies of interference in serial verbal reactions. *J. Exp. Psychol.* **18**, 643–662 (1935).

20. Mathiowetz, V., Weber, K., Kashman, N. & Volland, G. Adult Norms For The Nine Hole Peg Test Of Finger Dexterity. *Occup. Ther. J. Res.* **5**, 24 (1985).

21. Dziuban, C. D. & Shirkey, E. C. When is a correlation matrix appropriate for factor analysis? Some decision rules. *Psychol. Bull.* **81**, 358–361 (1974).
